# Higher Mortality in Trauma Patients Is Associated with Stress-Induced Hyperglycemia, but Not Diabetic Hyperglycemia: A Cross-Sectional Analysis Based on a Propensity-Score Matching Approach

**DOI:** 10.3390/ijerph14101161

**Published:** 2017-09-30

**Authors:** Cheng-Shyuan Rau, Shao-Chun Wu, Yi-Chun Chen, Peng-Chen Chien, Hsiao-Yun Hsieh, Pao-Jen Kuo, Ching-Hua Hsieh

**Affiliations:** 1 Department of Neurosurgery, Kaohsiung Chang Gung Memorial Hospital and Chang Gung University College of Medicine, Kaohsiung City 833, Taiwan; ersh2127@cloud.cgmh.org.tw; 2 Department of Anesthesiology, Kaohsiung Chang Gung Memorial Hospital and Chang Gung University College of Medicine, Kaohsiung City 833, Taiwan; shaochunwu@gmail.com; 3 Department of Plastic Surgery, Kaohsiung Chang Gung Memorial Hospital and Chang Gung University College of Medicine, Kaohsiung City 833, Taiwan; libe320@yahoo.com.tw (Y.-C.C.); VENU_CHIEN@hotmail.com (P.-C.C.); sylvia19870714@hotmail.com (H.-Y.H.)

**Keywords:** stress-induced hyperglycemia, diabetic hyperglycemia, diabetes mellitus, mortality

## Abstract

*Background:* Stress-induced hyperglycemia (SIH) is a form of hyperglycemia secondary to stress and commonly occurs in patients with trauma. Trauma patients with SIH have been reported to have an increased risk of mortality. However, information regarding whether these trauma patients with SIH represent a distinct group with differential outcomes when compared to those with diabetic hyperglycemia (DH) remains limited. *Methods:* Diabetes mellitus (DM) was determined by patient history and/or admission glycated hemoglobin (HbA1c) ≥6.5%. Non-diabetic normoglycemia (NDN) was determined by a serum glucose level <200 mg/dL in the patients without DM. Diabetic normoglycemia (DN) was determined by a serum glucose level <200 mg/dL in the patients with DM. DH and SIH was diagnosed by a serum glucose level ≥200 mg/dL in the patients with and without DM, respectively. Detailed data of these four groups of hospitalized patients, which included NDN (*n* = 7806), DN (*n* = 950), SIH (*n* = 493), and DH (*n* = 897), were retrieved from the Trauma Registry System at a level I trauma center between 1 January 2009 and 31 December 2015. Patients with incomplete registered data were excluded. Categorical data were compared with Pearson chi-square tests or two-sided Fisher exact tests. The unpaired Student’s *t*-test and the Mann–Whitney *U*-test were used to analyze normally distributed continuous data and non-normally distributed data, respectively. Propensity-score-matched cohorts in a 1:1 ratio were allocated using NCSS software with logistic regression to evaluate the effect of SIH and DH on the outcomes of patients. *Results:* The SIH (median [interquartile range: Q1–Q3], 13 [9–24]) demonstrated a significantly higher Injury Severity Score (ISS) than NDN (9 [4–10]), DN (9 [4–9]), and DH (9 [5–13]). SIH and DH had a 12.3-fold (95% confidence interval [CI] 9.31–16.14; *p* < 0.001) and 2.4-fold (95% CI 1.71–3.45; *p* < 0.001) higher odds of mortality, respectively, when compared to NDN. However, in the selected propensity-score-matched patient population, SIH had a 3.0-fold higher odd ratio of mortality (95% CI 1.96–4.49; *p* < 0.001) than NDN, but DH did not have a significantly higher mortality (odds ratio 1.2, 95% CI 0.99–1.38; *p* = 0.065). In addition, SIH had 2.4-fold higher odds of mortality (95% CI 1.46–4.04; *p* = 0.001) than DH. These results suggest that the characteristics and injury severity of the trauma patients contributed to the higher mortality of these patients with hyperglycemia upon admission, and that the pathophysiological effect of SIH was different from that of DH. *Conclusions:* Although there were worse mortality outcomes among trauma patients presenting with hyperglycemia, this effect was only seen in patients with SIH, but not DH when controlling for age, sex, pre-existed co-morbidities, and ISS.

## 1. Background

Stress-induced hyperglycemia (SIH) is a form of hyperglycemia secondary to stress and it commonly occurs in patients with critical illnesses such as trauma [1,2,3,4,5,6,7], burn injuries [8], myocardial infarction [9,10], stroke [11,12], and sepsis [13]. The neuroendocrine response to stress can result in up to 10 times greater adrenal cortical output, which is characterized by excessive gluconeogenesis, glycogenolysis, and insulin resistance [14]. The pathophysiology of SIH is thought to reflect temporary insulin resistance and relative insulin deficiency. The insulin resistance is driven by the overwhelming activation of pro-inflammatory mediators (tumor necrosis factor-α and interleukin-6) and counter-regulatory hormone excesses (glucagon, cortisol, and catecholamines) [15]. In such metabolic milieus, insulin fails to suppress hepatic gluconeogenesis despite the presence of hyperglycemia and the insulin-mediated glucose uptake into skeletal muscle is impaired [16]. Furthermore, the insulin concentrations in the plasma are inadequate to compensate for hyperglycemia [17].

A positive correlation was observed between admission hyperglycemia, and morbidity and mortality in trauma patients [2,3,7,18]. In a retrospective evaluation of 6099 surgical patients who had received vascular, gastrointestinal, liver transplants, trauma, and other miscellaneous surgeries, a relationship between hyperglycemia and mortality depending on the reason for admission was identified [1]. Among these patients, trauma patients with hyperglycemia had a more pronounced mortality than any other type of surgical patients [1]. In addition, published studies have consistently shown higher morbidity and mortality rates in the trauma patients with SIH [3,6,7,11,19]. However, few studies have evaluated the differential effect of SIH versus diabetic hyperglycemia (DH) on the outcomes of the trauma population. It has been reported that SIH, but not DH was associated with a significantly higher mortality risk in trauma patients [20]. Patients with SIH demonstrated a >2-fold increase in mortality risk (relative risk (RR) 2.41, 95% confidence interval (CI) 1.81–3.23) when compared to patients with DH who had a non-significant increase in their mortality risk (RR 1.47, 95% CI 0.92–2.36), [20]. So far, information regarding whether these trauma patients with SIH represent a distinct group with differential outcomes in comparison with those with DH remains limited. In this study, we aimed to assess the effect of SIH and DH on the outcomes of trauma patients. Propensity-score-matched patients were selected to reduce the effect of differences of sex and age, pre-existed co-morbidities, and injury severity to the patient population on the outcome assessment. The primary hypothesis of this study was that patients with SIH had a similar worse outcome as those patients with DH.

## 2. Methods

### 2.1. Ethics Statement

The Institutional Review Board (IRB) of the Kaohsiung Chang Gung Memorial Hospital, a Level I regional trauma center in Southern Taiwan [21,22], approved this study (reference number 201600006B0). Informed consent was waived according to IRB regulations.

### 2.2. Data Source and Study Population

**Figure 1:**
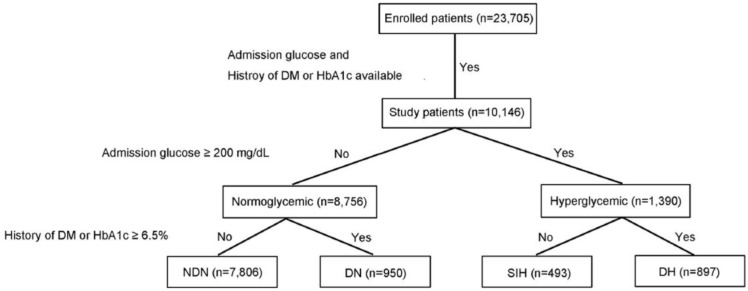
Flow chart of allocating patients into groups of non-diabetic normoglycemia (NDN), diabetic normoglycemia (DN), stress-induced hyperglycemia (SIH), and diabetic hyperglycemia (DH).

This retrospective study reviewed the data of all adult hospitalized trauma patients registered in the Trauma Registry System of the hospital from 1 January 2009 to 31 December 2015. Only adult patients where *t* ≥ 20 years old with available data regarding serum glucose level, a history of diabetes mellitus (DM), or a glycated hemoglobin (HbA1c) level were included in the study. Those patients with incomplete data were excluded. Hyperglycemia was defined as a serum glucose level ≥ 200 mg/dL upon arrival at the emergency department, which has been previously used by several trauma studies as a commonly utilized cutoff to define hyperglycemia. DM was determined by patient history and/or admission HbA1c ≥6.5%, based on current recommendations for the DM diagnosis from American Diabetes Association [23]. Non-diabetic normoglycemia (NDN) was determined by a serum glucose level <200 mg/dL in the patients without DM. DN was determined by a serum glucose level < 200 mg/dL in the patients with DM. DH and SIH was diagnosed by a serum glucose level ≥200 mg/dL in the patients with and without DM, respectively. Based on these definitions, the study patients were allocated into four exclusive groups (Figure 1). Detailed patient information retrieved from the Trauma Registry System included the following: age; sex; co-morbidities, such as hypertension (HTN), coronary artery disease (CAD), congestive heart failure (CHF), cerebral vascular accident (CVA), and end-stage renal disease (ESRD); injury severity score (ISS), which is expressed as the median and interquartile range (IQR, Q1–Q3); serum glucose level at the emergency department; HbA1c level; morbidity such as pneumonia or acute renal failure diagnosed after the admission; hospital length of stay (LOS); the rates of admission into the intensive care unit (ICU); in-hospital mortality.

### 2.3. Statistical Analysis

IBM SPSS Statistics for Windows, version 20.0 (IBM Corp., Armonk, NY, USA) was used for the statistical analysis. The primary outcome of the study was in-hospital mortality. The secondary outcomes were hospital LOS and ICU admission rate as well as rates of pneumonia and acute renal failure. Odds ratios (ORs) with 95% CIs of the associated conditions of the patients were presented. Two-sided Fisher exact or Pearson chi-square tests were used to compare categorical data. The unpaired Student’s *t*-test and Mann-Whitney *U*-test were used to analyze normally distributed continuous and non-normally distributed data, respectively, which was reported as mean ± standard deviation. To minimize confounding effects of sex and age, pre-existed co-morbidities, and injury severity of the patient population, propensity-score-matched groups of patients were selected for the assessment of the effect of SIH and DH on the outcomes. A logistic regression model was used to calculate the propensity scores with the following covariates: sex, age, co-morbidities, and ISS. A 1:1 matched study group was created by the Greedy method with a 0.2 caliper width using NCSS 10 software (NCSS Statistical software, Kaysville, UT, USA). After adjustment of these confounding factors, binary logistic regression was used for evaluating the effect of SIH and DH on the primary and secondary outcomes. *p*-values < 0.05 were defined as statistically significant.

## 3. Results

### 3.1. Characteristics of the Patients

As shown in Figure 1 and Table 1, the enrolled study population included 10,146 patients, who were allocated into four groups: NDN (*n* = 7,806), DN (*n* = 950), SIH (*n* = 493), and DH (*n* = 897). Compared to NDN, no significant difference in sex was found for SIH, but there was a significant female predominance in the DN and DH groups (Table 2). The patients of SIH as well as the DN and DH groups were significantly older than those in the NDN group. DN and DH had significantly higher rates of pre-existed co-morbidities than NDN. However, only higher odds of HTN, but not other co-morbidities, were found in SIH compared to NDN. The SIH (median [IQR: Q1–Q3], 13 [9–24]) had a remarkable significantly higher ISS than the NDN (9 [4–10]), DN (9 [4–9]), and DH (9 [5–13]). In addition, more patients in the SIH had an ISS of 16–24 and an ISS ≥25, but fewer patients had an ISS < 16 when compared to the NDN. Compared to DH, there was a significant female predominance, a younger age, higher rates of pre-existed co-morbidities (HTN, CAD, CHF, and CVA), and higher ISS in the SIH. 

### 3.2. Outcomes of the Patients

Compared to the NDN, the SIH had a 12.3-fold higher odds of mortality (95% CI 9.31–16.14; *p* < 0.001), a significantly longer hospital LOS (14.1 days vs. 9.6 days, respectively; *p* < 0.001), a higher proportion of patients admitted to the ICU (46.5% vs. 20.0%, respectively; *p* < 0.001), a 2.9-fold higher odds of pneumonia (95% CI 1.68–4.93; *p* < 0.001), and a 4.8-fold higher odds of acute renal failure (95% CI 2.15–10.52; *p* < 0.001). DH had 2.4-fold higher odds of mortality (95% CI 1.71–3.45; *p* < 0.001), a longer hospital LOS (12.1 days vs. 9.6 days, respectively; *p* < 0.001), a higher proportion of patients were admitted to the ICU (24.3% vs. 20.0%, respectively; *p* = 0.002), and higher odds of patients experiencing pneumonia-induced complications (OR 2.6; 95% CI 1.65–3.98; *p* < 0.001). However, the DH group did not demonstrate any acute renal failure. Although the DN did not have significantly higher mortality or a higher proportion of patients admitted to the ICU when compared to the NDN, the former had a longer hospital LOS and complication caused by pneumonia and acute renal failure. Compared to DH, SIH had a longer hospital LOS (14.1 days vs. 12.1 days, respectively; *p* < 0.001) as well as higher odds of patients experiencing pneumonia (OR 2.9; 95% CI 1.91–4.38; *p* < 0.001) and acute renal failure (OR 4.0; 95% CI 2.06–7.77; *p* < 0.001). However, the SIH did not have significantly higher mortality or a higher proportion of patients admitted to the ICU when compared to the DH.

### 3.3. Adjusted Outcomes of the Propensity Score-Matched Patients

Propensity-score-matched patients were selected to reduce the effect of differences of sex and age, pre-existed co-morbidities, and injury severity of the patient population on the outcome assessment (Table 3). Using NDN as the control, there were 934, 485, and 883 selected well-balanced pairs of DN, SIH, and DH, respectively. Using DH as the control, there were 366 selected well-balanced pairs of SIH. Among these selected pairs of patients, there were no significant differences in sex, age, co-morbidity, or ISS (Supplementary Materials Table S1). The logistic regression analysis of these pairs of patients showed that SIH had 3.0-fold higher odds of mortality (95% CI 1.96–4.49; *p* < 0.001), a significantly longer hospital LOS (14.1 days vs. 11.0 days, respectively; *p* = 0.016), and a significantly higher proportion of patients were admitted to the ICU (45.6% vs. 40.0%, respectively; *p* = 0.014) than NDN, but there were no differences in the rates of pneumonia and acute renal failure between SIH and NDN. However, although DH still had a longer hospital stay than NDN (12.0 days vs. 10.1 days, respectively; *p* = 0.016), DH did not have a significantly higher mortality (OR 1.2; 95% CI 0.99–1.38; *p* = 0.065), proportion of patients admitted to the ICU, or rates of pneumonia and acute renal failure than NDN. Compared to NDN, DN did not have a significantly higher mortality or proportion of patients that were admitted to the ICU, and DN still had a longer hospital LOS and were complicated with significantly higher rates of acute renal failure. However, there was no difference in the rates of pneumonia between DN and NDN. Compared to DH, SIH had 2.4-fold higher odds of mortality (95% CI 1.46–4.04; *p* = 0.001) and a significantly higher proportion of patients were admitted to the ICU (35.5% vs. 30.3%, respectively; *p* = 0.046). However, there were no differences in the hospital LOS, rates of pneumonia and acute renal failure between SIH and DH.

## 4. Discussion

This study compared the clinical outcomes in a broad group of hospitalized hyperglycemia patients and found that SIH patients presented with significantly higher mortality than NDN patients. More importantly, after the adjustment of the differences of demographic characteristics, co-morbidities, and injury severity of the trauma patients, there was still a significantly higher mortality in the selected propensity-score-matched SIH patients, but not in the DH patients. Notably, a large injury burden indicated by a high ISS is a typical risk factor for SIH in acute trauma patients [24], and in this study the SIH group had a remarkable significantly higher ISS than NDN and even the DH. Therefore, adjustment of the injury severity as well as possible confounding factors of the studied patient population is mandatory before assessment of the outcome. In this study, compared with NDN, SIH and DH had 12.3-fold and 2.4-fold higher odds of mortality, respectively. However, after adjustment of the confounding factors, including sex and age, pre-existing co-morbidities, and injury severity, although SIH had 3.0-fold higher odds of mortality than NDN, the same was not observed for DH who did not present significantly higher mortality odds. This result was in accordance with that reported by Kerby et al. [20], who demonstrated that the adjusted mortality remained significant for patients with SIH, but the mortality risk in diabetics was not significant when adjusted for age, sex, injury mechanism, and ISS. This result suggested that characteristics and injury severity of the trauma patients contributed to the higher mortality of these patients with admission hyperglycemia and the pathophysiological effect associated with SIH was different than that of DH. In this study, SIH had 2.4-fold higher adjusted odds of mortality than DH. The mechanism for potential detrimental effects in these two hyperglycemia states differs in their effect on outcomes after trauma. SIH is an acute process initiated by stress hormone and cytokine release secondary to stress; in contrast, DH is a chronic process associated with prolonged hyperglycemia over time, which subsequently leads to microvascular changes and is manifested as coronary artery disease, nephropathy, and peripheral vascular disease [19]. 

There is a large body of literature detailing the deleterious effects of hyperglycemia on immune system function [25]. An admission glucose level >200 mg/dL was also a predictor of infectious complications in the form of pneumonia, urinary tract infections, wound infections, and bacteremia [2]. Mean perioperative glucose levels greater than 220 mg/dL were associated with a seven times higher risk of infection in orthopedic trauma patients with no known history of diabetes mellitus [26]. SIH was associated with surgical site infections [4,5,6]. In a prospective observational cohort of stable nondiabetic patients with orthopedic injuries, 20% of patients were identified as SIH and were more likely to develop surgical site infections [27]. In addition, it had been reported that there was no higher risk of pneumonia in the patients with SIH (RR 1.44, 95% CI 1.08–1.93) and their risk of pneumonia was similar to those with DH (RR 1.49, 95% CI 1.03–2.17) [15]. 

Alveolar macrophages from normal hosts demonstrate an aberrant immune response that contributes to worsening infection and autoimmunity when exposed to elevated glucose concentration [28]. Insulin resistance was detected two days prior to the clinical suspicion of ventilator-associated pneumonia for critically injured patients on the glycemic control protocol [29]. However, in this study, although higher odds of pneumonia were found in the SIH and DH than in the NDN, this difference was not significant after the adjustment of the confounding factors, indicating that the higher risk of pneumonia in these trauma patients may be mostly attributed to their associated personal characteristics and injury severity.

Furthermore, in this study, SIH had 4.8-fold higher odds of acute renal failure than NDN, but the increased risk of acute renal failure, albeit high, was not significant in the propensity-score-matched patient population after the adjustment of the confounding factors. In addition, the risk of acute renal failure was not significantly higher in DN than NDN, regardless of the patient cohort before or after matching. However, these observations were less conclusive when considering that there was a lack of certain critical factors in this study, which included, albeit not limited to, the blood pressure, pre-injured renal function, and the therapeutic reagents used.

There were some other limitations in this study that should be acknowledged. First, the patients declared dead at the scene of accident or on hospital arrival were not included in the database, and this may have led to a selection bias. Second, the retrospective design of the study may create an inherent selection bias. Third, SIH and DH are not mutually exclusive entities, as DH patients may have some degree of stress response invoking their hyperglycemia. Because we did not measure stress response hormones or catecholamine levels, it was not possible for us to specifically identify whether stress might be more responsible for the hyperglycemia in the patients with DH. In addition, certain confounding factors such as the type of injury (penetrating, or burn), traumatic brain injury, and admission physiologic parameter (change of base excess), which were more related to the occurrence of SIH and the mortality, were not included as variables in this study for propensity score matching. This may result in an over-estimation of the mortality outcome for SIH. Furthermore, we did not collect and analyze data on glucose levels after the initial value in the emergency department. Although the use of glycemic control in critically ill trauma patients has been inconclusive as to whether tight glycemic control improves morbidity and mortality, we could only rely on the assumption of uniform management of these patients. This also indicates that defining appropriate treatment of SIH remains an area of ongoing investigation.

## 5. Conclusions

Although this study supports previous studies showing worse outcomes with respect to mortality among trauma patients presenting with hyperglycemia, this effect was only seen in patients with SIH and not in those with DH after controlling for age, sex, pre-existed co-morbidities, and ISS.

## Figures and Tables

**Table 1:** Characteristics, injury severities, and outcomes of the patients.

Variables	NDN (*n* = 7806)	DN (*n* = 950)	SIH (*n* = 493)	DH (*n* = 897)
Sex				
Male	4605 (59.0)	436 (45.9)	287 (58.2)	388 (43.3)
Female	3201 (41.0)	514 (54.1)	206 (41.8)	509 (56.7)
Age	51.7 ± 19.3	67.9 ± 12.3	56.7 ± 18.0	65.8 ± 12.4
Comorbidity				
HTN	1697 (21.7)	624 (65.7)	128 (26.0)	544 (60.6)
CAD	207 (2.7)	92 (9.7)	15 (3.0)	87 (9.7)
CHF	54 (0.7)	20 (2.1)	3 (0.6)	23 (2.6)
CVA	224 (2.9)	113 (11.9)	11 (2.2)	83 (9.3)
ESRD	7 (0.1)	1 (0.1)	0 (0.0)	3 (0.3)
ISS, median (IQR)	9 (4–10)	9 (4–9)	13 (9–24)	9 (5–13)
<16	6483 (83.1)	797 (83.9)	272 (55.2)	700 (78.0)
16–24	953 (12.2)	114 (12.0)	104 (21.1)	134 (14.9)
≥25	370 (4.7)	39 (4.1)	117 (23.7)	63 (7.0)
Mortality, *n* (%)	151 (1.9)	24 (2.5)	96 (19.5)	41 (4.6)
Hospital LOS (days)	9.6 ± 10.1	10.7 ± 10.5	14.1 ± 16.7	12.1 ± 11.8
ICU admission, *n* (%)	1560 (20.0)	213 (22.4)	229 (46.5)	218 (24.3)
Pneumonia	90 (1.2)	31 (3.3)	16 (3.2)	26 (2.9)
Acute renal failure	27 (0.3)	13 (1.4)	8 (1.6)	7 (0.8)

CAD = coronary artery disease; CHF = Congestive Heart Failure; CVA = cerebral vascular accident; DH = diabetic hyperglycemia; DN = diabetic normoglycemia; HTN = hypertension; ICU = intensive care unit; IQR = interquartile range; ISS = injury severity score; LOS = length of stay; NDN = nondiabetic normoglycemia; OR = odds ratio; SIH = stress-induced hyperglycemia.

**Table 2:** Comparison of the characteristics, injury severities, and outcomes among the patient groups.

Variables	DN vs. NDN		SIH vs. NDN		DH vs. NDN		SIH vs. DH	
	OR (95% CI)	*p*	OR (95% CI)	*p*	OR (95% CI)	*p*	OR (95% CI)	*p*
Sex		<0.001		0.733		<0.001		<0.001
Male	0.6 (0.52–0.68)		1.0 (0.81–1.17)		0.5 (0.46–0.61)		0.6 (0.52–0.68)	
Female	1.7 (1.48–1.94)		1.0 (0.86–1.24)		1.9 (1.64–2.17)		1.7 (1.48–1.94)	
Age	-	<0.001	-	<0.001	-	<0.001	-	<0.001
Comorbidity								
HTN	6.9 (5.96–7.96)	<0.001	1.3 (1.03–1.56)	0.028	5.5 (4.80–6.41)	<0.001	6.9 (5.96–7.96)	<0.001
CAD	3.9 (3.05–5.08)	<0.001	1.2 (0.68–1.96)	0.602	3.9 (3.04–5.12)	<0.001	3.9 (3.05–5.08)	<0.001
CHF	3.1 (1.84–5.18)	<0.001	0.9 (0.27–2.82)	1.000	3.8 (2.31–6.19)	<0.001	3.1 (1.84–5.18)	<0.001
CVA	4.6 (3.61–5.79)	<0.001	0.8 (0.42–1.43)	0.407	3.5 (2.66–4.49)	<0.001	4.6 (3.61–5.79)	<0.001
ESRD	1.2 (0.14–9.55)	0.601	-	1.000	3.7 (0.97–14.48)	0.075	1.2 (0.14–9.55)	0.601
ISS, median (IQR)	-	<0.001	-	<0.001	-	<0.001	-	<0.001
<16	1.1 (0.89–1.28)	0.512	0.3 (0.21–0.30)	<0.001	0.7 (0.61–0.86)	<0.001	1.1 (0.89–1.28)	0.512
16–24	1.0 (0.80–1.21)	0.853	1.9 (1.53–2.41)	<0.001	1.3 (1.04–1.54)	0.019	1.0 (0.80–1.21)	0.853
≥25	0.9 (0.61–1.21)	0.381	6.3 (4.96–7.89)	<0.001	1.5 (1.15–2.00)	0.003	0.9 (0.61–1.21)	0.381
Mortality, *n* (%)	1.3 (0.85–2.03)	0.218	12.3 (9.31–16.14)	<0.001	2.4 (1.71–3.45)	<0.001	1.3 (0.85–2.03)	0.218
Hospital LOS (days)	-	0.004	-	<0.001	-	<0.001	-	0.004
ICU admission, *n* (%)	1.2 (0.98–1.36)	0.078	3.5 (2.89–4.18)	<0.001	1.3 (1.09–1.51)	0.002	1.2 (0.98–1.36)	0.078
Pneumonia	2.9 (1.91–4.38)	<0.001	2.9 (1.68–4.93)	<0.001	2.6 (1.65–3.98)	<0.001	2.9 (1.91–4.38)	<0.001
Acute renal failure	4.0 (2.06–7.77)	<0.001	4.8 (2.15–10.52)	0.001	2.3 (0.98–5.22)	0.079	4.0 (2.06–7.77)	<0.001

CAD = coronary artery disease; CHF = Congestive Heart Failure; CI = confidence interval; CVA = cerebral vascular accident; DH = diabetic hyperglycemia; DN = diabetic normoglycemia; HTN = hypertension; ICU = intensive care unit; IQR = interquartile range; ISS = injury severity score; LOS = length of stay; NDN = nondiabetic normoglycemia; OR = odds ratio; SIH = stress-induced hyperglycemia.

**Table 3:** Comparison of the outcomes in the selected propensity-score-matched cohort.

Propensity-Score-Matched Cohort				
**DN vs. NDN**	**DN (*n* = 934)**	**NDN (*n* = 934)**	**OR (95% CI)**	***p***
Mortality, *n* (%)	23 (2.5)	21 (2.2)	1.1 (0.61–1.98)	0.763
Hospital LOS (days)	10.6 ± 10.5	9.4 ± 9.4	-	0.010
ICU admission, *n* (%)	207 (22.2)	185 (19.8)	1.3 (0.95–1.69)	0.109
Pneumonia	30 (3.2)	17 (1.8)	1.8 (0.98–3.34)	0.056
Acute renal failure	11 (1.2)	2 (0.2)	5.5 (1.22–24.81)	0.027
**SIH vs. NDN**	**SIH (*n* = 485)**	**NDN (*n* = 485)**	**OR (95% CI)**	***p***
Mortality, *n* (%)	89 (18.4)	30 (6.2)	3.0 (1.96–4.49)	<0.001
Hospital LOS (days)	14.1 ± 16.6	11.0 ± 14.9	-	0.016
ICU admission, *n* (%)	221 (45.6)	194 (40.0)	1.6 (1.10–2.30)	0.014
Pneumonia	15 (3.1)	11 (2.3)	1.4 (0.62–3.15)	0.416
Acute renal failure	6 (1.2)	1 (0.2)	6.0 (0.72–49.84)	0.097
**DH vs. NDN**	**DH (*n* = 883)**	**NDN (*n* = 883)**	**OR (95% CI)**	***p***
Mortality, *n* (%)	40 (4.5)	25 (2.8)	1.2 (0.99–1.38)	0.065
Hospital LOS (days)	12.0 ± 11.8	10.1 ± 10.1	-	<0.001
ICU admission, *n* (%)	214 (24.2)	206 (23.3)	1.1 (0.82–1.47)	0.547
Pneumonia	26 (2.9)	19 (2.2)	1.4 (0.76–2.47)	0.299
Acute renal failure	7 (0.8)	4 (0.5)	1.8 (0.51–5.98)	0.372
**SIH vs. DH**	**SIH (*n* = 366)**	**DH (*n* = 366)**	**OR (95% CI)**	***p***
Mortality, *n* (%)	51 (13.9)	21 (5.7)	2.4 (1.46–4.04)	0.001
Hospital LOS (days)	12.7 ± 16.0	12.6 ± 13.1	-	0.872
ICU admission, *n* (%)	130 (35.5)	111 (30.3)	1.5 (1.01–2.36)	0.046
Pneumonia	8 (2.2)	13 (3.6)	0.6 (0.26–1.49)	0.280
Acute renal failure	3 (0.8)	4 (1.1)	0.8 (0.17–3.35)	0.706

## Supplementary Materials

The following are available online at www.mdpi.com/1660-4601/14/10/1161/s1. Table S1: Covariates and the outcome assessment of polytrauma and non-polytrauma patients adjusted in 1:1 greedy propensity-score matching.
