# Supplementary material for: Higher Mortality in Trauma Patients Is Associated with Stress-Induced Hyperglycemia, but Not Diabetic Hyperglycemia: A Cross-Sectional Analysis Based on a Propensity-Score Matching Approach
Source: Int J Environ Res Public Health. 2017 Sep 30;14(10):1161. doi: 10.3390/ijerph14101161 (PMC5664662; doi:10.3390/ijerph14101161)
Supplement: Supplementary file 1 [file ijerph-14-01161-s001.pdf]

Higher Mortality in Trauma Patients Is Associated with Stress-Induced Hyperglycemia, but Not Diabetic Hyperglycemia: A Cross-Sectional Analysis Based on a Propensity-Score Matching Approach

Cheng-Shyuan Rau, Shao-Chun Wu, Yi-Chun Chen, Peng-Chen Chien, Hsiao-Yun Hsieh, Pao-Jen Kuo and Ching-Hua Hsieh

Table S1. Covariates and the outcome assessment of polytrauma and non-polytrauma patients adjusted in 1:1 greedy propensity-score matching.

	Propensity-Score-Matched Cohort			
**DN vs. NDN**	**DN (*n* = 934)**	**NDN (*n* = 934)**	**OR (95% CI)**	***p***
Sex				1.000
Male	427 (45.7)	427 (45.7)	1.0 (0.83–1.20)	
Female	507 (54.3)	507 (54.3)	1.0 (0.83–1.20)	
Age	67.9 ± 12.3	67.9 ± 12.2	-	0.986
Comorbidity				
HTN	609 (65.2)	609 (65.2)	1.0 (0.83–1.21)	1.000
CAD	79 (8.5)	79 (8.5)	1.0 (0.72–1.39)	1.000
CHF	13 (1.4)	13 (1.4)	1.0 (0.46–2.17)	1.000
CVA	103 (11.0)	103 (11.0)	1.0 (0.75–1.34)	1.000
ESRD	0 (0.0)	0 (0.0)	-	-
ISS, median (IQR)	9 (4–9)	9 (4–9)	-	0.996
**SIH vs. NDN**	**SIH (*n* = 485)**	**NDN (*n* = 485)**	**OR (95% CI)**	***p***
Sex				1.000
Male	284 (58.6)	284 (58.6)	1.0 (0.78–1.29)	
Female	201 (41.4)	201 (41.4)	1.0 (0.78–1.29)	
Age	56.6 ± 17.9	56.8 ± 17.8	-	0.883
Comorbidity				
HTN	125 (25.8)	125 (25.8)	1.0 (0.75–1.33)	1.000
CAD	14 (2.9)	14 (2.9)	1.0 (0.47–2.12)	1.000
CHF	3 (0.6)	3 (0.6)	1.0 (0.20–4.98)	1.000
CVA	10 (2.1)	10 (2.1)	1.0 (0.41–2.43)	1.000
ESRD	0 (0.0)	0 (0.0)	-	-
ISS, median (IQR)	10 (9–22)	10 (9–22)	-	0.929
**DH vs. NDN**	**DH (*n* = 883)**	**NDN (*n* = 883)**	**OR (95% CI)**	***p***
Sex				1.000
Male	383 (43.4)	383 (43.4)	1.0 (0.83–1.21)	
Female	500 (56.6)	500 (56.6)	1.0 (0.83–1.21)	
Age	65.8 ± 12.4	65.9 ± 12.5	-	0.895
Comorbidity				
HTN	533 (60.4)	533 (60.4)	1.0 (0.83–1.21)	1.000
CAD	78 (8.8)	78 (8.8)	1.0 (0.72–1.39)	1.000
CHF	14 (1.6)	14 (1.6)	1.0 (0.47–2.11)	1.000
CVA	80 (9.1)	80 (9.1)	1.0 (0.72–1.38)	1.000
ESRD	1 (0.1)	1 (0.1)	1.0 (0.06–16.01)	1.000
ISS, median (IQR)	9 (5–13)	9 (5–12)	-	0.976
**SIH vs. DH**	**SIH (*n* = 366)**	**DH (*n* = 366)**	**OR (95% CI)**	***p***
Sex				1.000

Male	192 (52.5)	192 (52.5)	1.0 (0.75–1.34)	
Female	174 (47.5)	174 (47.5)	1.0 (0.75–1.34)	
Age	61.9 ± 15.0	61.9 ± 14.3	-	0.938
Comorbidity				
HTN	121 (33.1)	121 (33.1)	1.0 (0.74–1.36)	1.000
CAD	13 (3.6)	13 (3.6)	1.0 (0.46–2.19)	1.000
CHF	1 (0.3)	1 (0.3)	1.0 (0.06–16.05)	1.000
CVA	7 (1.9)	7 (1.9)	1.0 (0.35–2.88)	1.000
ESRD	0 (0.0)	0 (0.0)	-	-
ISS, median (IQR)	9 (4–16)	9 (5–16)	-	0.913

CAD = coronary artery disease; CHF = Congestive Heart Failure; CI = confidence interval; CVA = cerebral vascular accident; DH = diabetic hyperglycemia; DN = diabetic normoglycemia; HTN = hypertension; IQR = interquartile range; ISS = injury severity score; NDN = nondiabetic normoglycemia; OR = odds ratio; SIH = stress-induced hyperglycemia.

© 2017 by the authors. Licensee MDPI, Basel, Switzerland. This article is an open access article distributed under the terms and conditions of the Creative Commons Attribution (CC BY) license (http://creativecommons.org/licenses/by/4.0/).
